# Sex differences in risk factors, treatment, and prognosis in acute stroke

**DOI:** 10.1177/1745506520952039

**Published:** 2020-09-30

**Authors:** Solveig Dahl, Clara Hjalmarsson, Björn Andersson

**Affiliations:** 1 Department of Medicine, Sahlgrenska University Hospital, Sahlgrenska Academy, University of Gothenburg, Gothenburg, Sweden; 2 Department of Cardiology, Sahlgrenska University Hospital, Sahlgrenska Academy, University of Gothenburg, Gothenburg, Sweden

**Keywords:** differences, prognosis, sex, stroke, treatment

## Abstract

**Objectives:**

Stroke is a major cause of long-term disability and death worldwide. Several studies have shown that women in general have more severe symptoms at arrival to hospital and are less likely to return home and independent living. Our aim with the present study was to update previous results concerning sex differences in baseline characteristics, stroke management, and outcome in a population study from Sahlgrenska University Hospital, Gothenburg, Sweden.

**Methods:**

This study included patients with acute ischemic and hemorrhagic stroke in 2014 at Sahlgrenska University Hospital. All data were collected from The Swedish National Stroke Registry (Riksstroke).

**Results:**

The study population consisted of 1453 patients, with 46.7% females. Women were 5 years older than men. There was no sex difference in acute stroke severity. Frequency of revascularization was equal between men and women. The stroke mortality rate was the same between the sexes. At 3-months follow-up, women had a worse functional outcome and a higher frequency of depression and post-stroke fatigue.

**Conclusion:**

Our results show that there are no sex differences in management of acute stroke. However, the cause of worse functional outcome in women at 3-months follow-up, independent of other risk factors, is not clear and warrants further investigations.

## Introduction

Stroke is a major cause of long-term disability and death worldwide.^1,2^ Several studies have shown that the incidence of stroke is higher in men until advanced age, with a higher incidence of stroke in women after 85 years leading to an excess of disability and mortality in older women.^3^

Several studies have shown that women, in general, have more severe symptoms at arrival to hospital and that they are less likely to return home and to independent living.^4–8^ Some previous studies have suggested that women may be treated less aggressively for primary and secondary stroke prevention and for acute stroke, compared with men.^9,10^ However, recent studies on management of acute stroke care have demonstrated that there are no sex differences in rate of thrombolysis and thrombectomy in ischemic stroke (IS) and equal access to brain imaging, Holter monitoring, and stroke unit care.^11–14^ Sex differences have also recently been put forward in management of ischemic heart disease,^15^ STEMI (ST-segment-elevation myocardial infarction),^16^ heart failure,^17^ and in primary and secondary prevention of cardiovascular disease,^18^ which underline the significance of this issue.

Thus, whether sex affects aspects of stroke is not fully clarified, and present findings are not consistent. Better understanding of sex differences in stroke management and outcome may be important for health outcome. Therefore, our aim with the present study was to investigate possible differences between women and men in baseline characteristics, stroke management, and outcome in a hospital cohort with acute stroke patients.

## Methods

### Study sample

Patients with acute IS and intracerebral hemorrhage (ICH), who were admitted at Sahlgrenska University Hospital during 2014, were included. Patients with subarachnoid hemorrhage were excluded. All data were collected from The Swedish National Stroke Registry (Riksstroke), which has a coverage of nearly 90% of all in-hospital treated patients in Sweden.^19^ Estimates suggest that between 84% and 92% of patients with acute stroke are treated in hospitals in Sweden. The following demographic characteristics and comorbidities were collected: previous stroke, diabetes, hypertension, smoking, atrial fibrillation (AF), living alone at home, activities of daily living (ADL), as measured by mRS scores (modified Rankin Scale)^20^ and current medication use.

### Measures

The following in-hospital variables were included: time elapsed from stroke onset to arrival at hospital, NIHSS (National Institute of Health Stroke Scale),^21^ RLS (Reaction Level Scale)^22^ at admittance to hospital, revascularization or not, diagnostic procedures, stroke unit care, drugs prescribed at admittance and at discharge, and whether patients were discharged to home, rehabilitation clinic, or to an institution. Case fatality was registered at 27 and 90 days.

Three months after discharge, a validated questionnaire was sent to all living patients to collect information on ADL and symptoms of depression and fatigue. A second questionnaire was sent to non-responders and when information was still not obtained, patients were called by a skilled nurse.

### Variable definitions

Patients were defined as independent in ADL if they managed dressing, toilet visits, walking without assistance and if they were in, general, not dependent on assistance. Functional level at baseline was estimated by mRS, with scores ranging from 0 to 5. Patients with mRS = 0 have no symptoms; mRS = 1 able to carry out all usual activities, despite some minor symptoms; mRS = 2 slight disability, able to look after own affairs, but unable to carry out all previous activities; mRS ⩾ 3 means increasing disability, from unable to walk independently to entirely bedridden. According to a previous article,^23^ Riksstroke questions on ADL may be translated to mRS scores. However, it is not possible to separate mRS 0, 1, and 2 from each other. Therefore, these scores were clustered to 0–2.

RLS was defined as 1 = conscious 0 = unconscious. NIHSS scores 0–38, where 0 is defined as no neurological symptoms and a score of 38 means no reactions at all.

#### Statistical methods

Data are presented as percentage or mean ± standard deviation (SD) when applicable. We used non-parametric tests for comparing mean values of continuous variables, while the chi-square test was used for discrete variables. Spearman’s correlation coefficient was used for univariate correlations. Variables with a correlation (p ⩾ 0.15) to female gender were not included in binary logistic regression since they were not statistically significant.

The binary logistic regression was adjusted for the following confounders: age, NIHSS at hospital admittance, consciousness, a history of previous stroke, AF, diabetes, pre-stroke mRS, mRS at 3-months follow-up, living alone at 3-months follow-up, and post-stroke depression. The results are presented as odds ratio (OR) with 95% confidence intervals (CIs). P-values <0.05 were regarded as statistically significant (2-sided test). All statistical analyses were performed using the SPSS 23.0 package (SPSS Inc., Chicago, Illinois, USA).

#### Ethics

The study was approved by the Ethical Committee of Sahlgrenska University Hospital, Gothenburg University, Gothenburg 2015. Registration no.: 256-15.

## Results

### Baseline characteristics

Data were collected from Riksstroke for 1453 patients (Table 1), who were admitted to Sahlgrenska University Hospital in 2014 with IS or ICH. The study population consisted of 678 (46.7%) women and 775 men (53.3%, p = 0.01). Women were significantly older than men (78 ± 13 years vs 72 ± 13 years; p = 0.00).

**Table 1.** Baseline characteristics before stroke.

Variable	Males	Females	p-value^a^
Age, y (mean ± SD)	72 (13)	78 (13)	** 0.00**
Sex, n (%)	775 (53.3)	678 (46.7)	**0.01**
Living at home, n (%)	702 (91.3)	593 (87.7)	0.03
Living alone, n (%)	324 (42.3)	455 (67.7)	** 0.00**
mRS 0–2, n (%)	678 (90.0)	519 (78.5)	** 0.00**
Previous stroke, n (%)	200 (26.2)	140 (20.9)	0.02
Atrial fibrillation, n (%)	159 (20.7)	148 (22.1)	0.56
Diabetes, n (%)	181 (23.5)	102 (15.2)	** 0.00**
Hypertension, n (%)	419 (54.8)	381 (57)	0.39
Smokers, n (%)	111 (16.0)	79 (13.4)	0.21

y: years; SD: standard deviation; mRS: modified Rankin Scale.

Sample size for each variable may differ due to missing data in some patients.

a Non-parametric tests were used for comparing continuous variables, while chi-square test was used for discrete variables.

Compared with women, men had a higher rate of previous stroke (26.2% vs 20.9%, p = 0.02) and diabetes (23.5% vs 15.2%, p = 0.00). Prevalence of AF and hypertension was the same for both men and women. Before stroke, women were more often living alone (67.7% vs 42.2%, p = 0.00) and were less independent in ADL, as measured by mRS scores (mRS = 0–2; 78.5% vs 90.0%, p = 0.00).

### Hospital care

Data are presented in Table 2. In the Gothenburg area during 2014, 86% of all stroke patients were registered in Riksstroke. In total, 1226 (84.4%) of 1453 patients had IS, while 214 (14.7%) had ICH and in 13 patients (0.9%) stroke etiology was undetermined. Sex distribution was alike in patients with ICH, but more men than women had an IS. Stroke severity, as measured by NIHSS at hospital arrival, was similar between men and women.

**Table 2.** In-hospital care.

Variable	Men	Women	p-value^a^
N=	775 (53.3)	678	**0.01**
Intracerebral hemorrhagia, n (%)	**115 (53.7)**	**99**	**0.27**
Ischemic stroke	**655 (53.4)**	**571**	**0.02**
Time to hospital			
Arrival, hrs (mean, SD)	9 (19)	7 (12)	0.86
NIHSS, scores (mean, SD)	5 (6)	6 (7)	0.17
Unconscious at arrival, n (%)	120 (15.9)	125 (18.7)	0.16
Stroke unit care, n (%)	702 (91.3)	593 (87.7)	0.03
Thrombolysis, n (%)	55 (8.4)	49 (8.7)	0.92
Thrombectomy, n (%)	32 (4.9)	29 (5.1)	0.90
MRT, n (%)	184 (23.9)	109 (16.2)	**0.00**
CT, n (%)	753 (97.8)	664 (98.2)	0.58
Carotid ultrasound, n (%)	331 (44.0)	242 (36.7)	0.01
Holter monitoring, n (%)	546 (74.2)	428 (66.9)	0.00
Case fatality 27 days, n (%)	66 (9.7)	84 (14.0)	0.02
Discharge to home, n (%)	477 (69.7)	368 (62.3)	0.01
Discharge to a rehabilitation	52 (7.6)	44 (7.4)	1.00
Clinic, n (%)			
Discharge to municipal institution, n (%)	123 (18.0)	162 (27.4)	**0.00**

SD: standard deviation; hrs: hours; NIHSS: National Institute of Health Stroke Scale; MRT: magnetic resonance tomography; CT: computed tomography.

Sample size for each variable may differ due to missing data in some patients. Patients discharged to other hospital or other department are not included (male = 40 (4.7), women = 17 (2.9).

In 13 patients, stroke etiology was unknown.

a Non-parametric tests were used for comparing mean values of continuous variables, while chi-square test was used for discrete variables.

Furthermore, women were less often treated in a stroke unit (87.7% vs 91.3%, p = 0.03). Women were less likely to undergo magnetic resonance tomography (MRT, (16.2% vs 23.9%, p = 0.00)), carotid ultrasound (36.7% vs 44.0%, p = 0.01), and Holter monitoring (66.9% vs 74.2%, p = 0.00). Men and women were similarly treated, with respect to thrombolysis and thrombectomy. Case fatality at 27 days was higher in women than in men (14.0% vs 9.7%, p = 0.02).

After in-hospital care, fewer women than men were discharged to home (62.3% vs 69.7%, p = 0.01), and women ended-up more often at an institution (27.4% vs 18.0%, p = 0.00). Referral to a rehabilitation clinic did not differ between the sexes. A small number of males and females were referred to another department or to another hospital (4.7% vs 2.9%, not shown).

### Medical therapy

Before stroke, men were more often prescribed statins than women (32.1% vs 21.5%, p = 0.00), but there was no difference between the sexes, regarding prescribed antiplatelets or anticoagulants (Table 3).

**Table 3.** Drugs at hospital admittance and at discharge.

Variable	Before stroke		p-value^a^	At discharge		p-value^a^
	Men	Women		Men	Women	
Lipid lowering drugs, n (%)	243 (32.1)	142 (21.5)	**0.00**	485 (63.0)	362 (53.9)	0.00
Aspirin, n (%)	217 (28.7)	178 (27.0)	0.48	160 (23.1)	96 (16.3)	0.00
Clopidogrel, n (%)	59 (7.8)	40 (6.0)	0.21	343 (44.7)	285 (42.4)	0.40
Warfarin (AF and IS), n (%)	43 (32.8)	30 (24.2)	0.17	29 (26.1)	19 (18.3)	0.19
NOAC (AF and IS), n (%)	12 (9.2)	7 (5.6)	0.34	47 (43.1)	48 (47.1)	0.58

AF: atrial fibrillation; IS: ischemic stroke; NOAC: non-vitamin K oral anticoagulants.

Sample size for each variable may differ due to missing data in some patients.

a Chi-square test was used for discrete variables.

Even after hospital discharge, more men than women were prescribed statins (63.0% vs 53.9%, p = 0.00). In patients with AF and IS (n = 257), warfarin and NOAC (non-vitamin K oral anticoagulants) were prescribed equally to men and women; 67.3% of all patients with AF were treated with anticoagulants (not shown). At hospital discharge, men were prescribed aspirin more often than women (23.1% vs 16.3%, p = 0.00), but there was no sex difference in prescription of clopidogrel.

### Three-months follow-up

The 3-months questionnaire had an answer frequency of 85%. At 3-months follow-up (Table 4), 82.1% patients were living at home (not shown). More men than women lived at home (84.6% vs 79.2%, p = 0.03). Women were living alone more often than men (57.7% vs 37.7%, p = 0.00), they were more likely to be less independent (mRS 0–2; 54.2% vs 69.8%, p = 0.00), and had an increased frequency of depression (71.5% vs 59.3%, p = 0.00) and fatigue (91.1% vs 84.4%, p = 0.00) compared with men.

**Table 4.** Three-months follow-up.

Variable	Men	Women	p-value^a^
mRS (0–2), n (%)	386 (69.8)	249 (54.2)	**0.00**
Depression, n (%)	319 (59.3)	309 (71.5)	**0.00**
Treatment for depression, n (%)	132 (24.5)	111 (25.6)	0.71
Fatigue, n (%)	470 (84.4)	409 (91.1)	0.00
Living alone, n (%)	206 (37.7)	254 (57.7)	**0.00**
Living at home, n (%)	473 (84.6)	361 (79.2)	0.03
Case fatality 90 dy, n (%)	95 (14.2)	117 (19.9)	0.01

mRS: modified Rankin Scale; dy: days.

Sample size for each variable may differ due to missing data in some patients.

a Chi-square test was used for discrete variables.

Case fatality at 90 days was higher in women than in men (19.9% vs 14.2%, p = 0.01).

### Binary logistic regression

After adjustments for the aforementioned risk factors, discharge to home or to an institution was not different between women and men (not shown). Female sex (Table 5) was independently associated with worse mRS before stroke (OR, 2.05 (95% CI, 1.46–2.87)), and 3 months after stroke (OR, 1.50 (95% CI, 1.05–2.14)), depression at 3-months follow-up (OR, 1.67 (95% CI, 1.23–2.27)), and fatigue at 3-months follow-up (OR, 1.71, (95% CI, 1.14–2.58)).

**Table 5.** Binary logistic regression analysis showing the relationship between female sex and explanatory variables.

Variables	OR (CI)	p value
mRS before stroke	2.05 (1.46–2.87)	**0.00**
Stroke unit	1.44 (0.94–2.20)	0.09
mRS at 3-months follow-up	1.50 (1.05–2.14)	0.03
Depression at 3-months follow-up	1.67 (1.23–2.27)	0.00
Fatigue at 3-months follow-up	1.71 (1.14–2.58)	0.01
Mortality at 27 days	0.71 (0.35–1.46)	0.35
Mortality at 90 days	1.07 (0.61–1.85)	0.82

OR: odds ratio; CI: confidence interval; mRS: modified Rankin Scale (0–2 = independent in daily life; 3–5 = increasing disability, from unable to walk independently to entirely bedridden); NIHSS: National Institute of Health Stroke Scale; AF: atrial fibrillation.

Binary logistic regression adjusted for age, NIHSS at admittance, consciousness, AF, diabetes, a history of previous stroke, pre-stroke mRS, mRS at 3-months follow-up, living alone at 3-months follow-up, and post-stroke depression.

Sample size for each variable may differ due to missing data in some patients.

Case fatality at 27 and 90 days was not different between men and women after adjustment for depicted risk factors.

## Discussion

In the present study, men and women were treated alike, with regard to revascularization and admittance to stroke unit care. As for diagnostic procedures, more men than women underwent carotid ultrasound and Holter monitoring. Furthermore, long-term functional stroke outcome seemed to be worse in women. Our results are partly in agreement with several earlier reports^24–26^; however, in the present study, female sex alone seemed to be an independent risk factor for worse functional outcome, post-stroke depression, and fatigue, contradictory to previous reports.^27–29^

Case fatality after 27 days and after 90 days was the same in men and women, after adjustment for the aforementioned covariates. Women’s all-cause mortality has been put forward to be lower after adjustments for relevant risk factors in some reports^7,13^ but also, conversely, to be higher^24,30^ or similar between men and women,^8,31,32^ as in the present report. Variations in mortality between sexes may be caused by a disparity in the studied populations or may reflect differences in performed statistical adjustments.

In the present study, women were older than men and more often lived alone before the stroke, which may influence the functional stroke outcome in women.^33^ An increased prevalence of AF in women has consistently been demonstrated.^6,9,13,28^ This latter finding was not corroborated in the present study, which may partly explain the same stroke severity between men and women. Previous studies^28,30,31^ have shown that cases of cardio-embolic stroke are often more severe than those related to other causes.

### In-hospital stroke management

We found no sex differences in treatment with thrombolysis or thrombectomy during acute care. There was no difference in stroke outcome between males and females after revascularization (not shown). Earlier findings of worse functional 90-day outcome in women treated with revascularization,^14,34,35^ compared with men, may have been caused by notable differences in presentation characteristics, pre-hospital delay, older age, more severe strokes, and a higher rate of comorbidity in women. However, several recent studies have demonstrated a comparable functional outcome after thrombolysis or thrombectomy and greater years of optimal life in females compared with males.^11,12^ Our present findings are consistent with the latter studies.

Carotid imaging and Holter monitoring were performed less often in women in the present study in accordance with some prior studies.^10,28,31,36^ According to previous reports,^37,38^ fewer women undergo carotid revascularization, which may be explained by appropriate patient selection based on stroke characteristics, surgical eligibility, and the prevalence of severe carotid stenosis. However, despite these differences, National Guidelines in Stroke Care^39^ recommend that carotid ultrasound should be performed in all appropriate patients, independent of sex.

There is no obvious explanation for why carotid imaging and Holter monitoring were performed less often in the present study. It may be hypothesized that this may be caused by differences in age or comorbidities between men and women.

It has been reported that cardio-embolism is more frequent in women.^13,40^ However, this is not confirmed in the present study since we found no increased prevalence of AF in females before stroke.

More men than women were prescribed statins before stroke and a putative explanation for this may be the reported higher frequency of ischemic heart disease in men,^41^ although not explored in the present study. After hospital care, women were still less likely than men to be prescribed statins. A recent study^42^ showed that statins help prevent recurrent stroke in both sexes and therefore should be recommended, as a secondary prevention, to patients with IS. There is no obvious explanation for why statins are prescribed to a lesser degree to females in the present study. There were no sex differences in the use of anticoagulants in patients with AF, either before stroke or at discharge.

In some studies,^6,9,13^ more women than men were more likely to be discharged to institutions and less women discharged to home. These findings were not corroborated in the present study after adjustments for the aforementioned risk factors in spite of worse functional outcome in women. Municipal home-care is extended in Sweden, implying that even disabled patients may be able to stay at home.

### Three-months follow-up

The most intriguing finding in the present study was worse disability in women at 3-months follow-up, independent of age, previous stroke, AF, pre-stroke mRS, NIHSS at admittance, consciousness at admittance, living alone after stroke, and post-stroke depression. In agreement with our results, a higher dependency after stroke in women has previously been reported^8,13,24,25^ but has in several studies been associated with higher age and more severe stroke.^6,27,32^ However, in the present study, we have not identified any difference in management or treatment strategies in the acute phase that could serve to explain the worse outcome in women.

The clinical considerations in stroke outcome in men and women are likely multifactorial. In the present study, more women lived alone after stroke compared with men, which may have resulted in a lack of endorsement and counseling by the spouse, leading to less improvements in daily function. Yet, other factors may be important such as differences in biology between men and women, hormonal changes, differences in ischemic preconditioning, collateral circulation, increased systemic inflammation, alteration in gene expression, and neurological damage to the brain may have a different impact on women and men.^24,43^ Previous research also points to differences in muscle strength, which may be an important factor for functional outcome.^44^

In accordance with our present findings, depression after stroke is very common and more frequent in women.^45^ Post-stroke depression has convincingly been shown to affect long-term functional outcome,^46^ and in our study female sex was independently associated with post-stroke depression at 3-months follow-up. Persistent post-stroke depression at long-term follow-up may be associated with decreased level of pre-stroke social activities.^47^ In our study, more women than men were living alone before stroke, and more females had a lower level of functional activity, in accordance with previous findings.^24,33^ How-ever, we found no independent association between living alone prior to stroke and a higher level of disability and/or post-stroke depression (not shown). These differences in outcome may be due to population characteristics or largeness of the included population.

In the present report, more women than men claimed to have fatigue after 3-months follow-up. Previously, it has been demonstrated that there is a strong relationship between depression and post-stroke fatigue.^48^ The higher rate of this in women in the present study may be related to the notorious difficulty in differentiating between depression and fatigue in a specific patient.

In spite of higher frequency of depression in females in the present study, there was no difference between sexes in the use of antidepressants. It may be hypothesized that depression may be more elusive in women than in men and therefore less treated. Since untreated post-stroke depression may lead to a reduced quality of life and a lower level of functional recovery, it must be emphasized that all stroke patients should be routinely screened for depression.

Some of the limitations of this study are the retrospective design, which makes it difficult to draw any firm conclusions about cause-effect relationships; the use of administrative datasets, which may lead to misclassification biases, and the single-center design, suggesting that the population may not be representative of the stroke population in Sweden.

In conclusion, acute stroke treatment seems to be the same between sexes, but in the present study female sex alone seems to be an independent risk factor for worse outcome and post-stroke depression, which may limit the potential of long-term stroke rehabilitation in women. Better understanding of the type and degree of inequities between the sexes in stroke management and outcome is important for improving health outcomes; thus, further studies are warranted.
